# Prevalence, Age-Standardized Prevalence, and Incidence Rates of Bilateral High-Frequency Hearing Loss among Japanese Individuals Undergoing Comprehensive Health Checkup System (*Ningen Dock*) from 2014 to 2020: A Descriptive Study

**DOI:** 10.31662/jmaj.2024-0328

**Published:** 2025-12-19

**Authors:** Yuri Akamatsu, Yoshitaka Nishikawa, Mayumi Toyama, Yoshimitsu Takahashi, Hiroshi Nakanishi, Kiyoshi Misawa, Toshiyuki Ojima, Takeo Nakayama

**Affiliations:** 1 Department of Health Informatics, School of Public Health, Kyoto University, Kyoto, Japan; 2 Department of Community Health and Preventive Medicine, Hamamatsu University School of Medicine, Hamamatsu, Japan; 3 Department of Implementation Science in Public Health, Kyoto University School of Public Health, Kyoto, Japan; 4 Department of Otorhinolaryngology/Head & Neck Surgery, Hamamatsu University School of Medicine, Hamamatsu, Japan

**Keywords:** high-frequency hearing loss, prevalence, incidence rate, *Ningen Dock*

## Abstract

**Introduction:**

Hearing loss is a public concern, considering its high prevalence and negative effects on older adults. Limited data on hearing loss are available from Japan, which has a high aging rate. Hearing loss generally begins bilaterally at high frequencies with age. This study aimed to describe the prevalence, age-standardized prevalence, and incidence rates of bilateral high-frequency hearing loss (HFHL), using data from Japan.

**Methods:**

This descriptive study utilized *Ningen Dock* and regular health check-up examination data obtained from the Seirei Health Care Division from 2014 to 2020. The outcome was bilateral inaudibility of 40 dB at 4 kHz (bilateral HFHL). The prevalence in each age group in 2020, age-standardized prevalence from 2014 to 2020 using the direct method, and incidence rates (per 1,000 person-years) were calculated by sex.

**Results:**

Most participants (60% male) underwent *Ningen Dock*. Each year, the number of participants was 55,000-62,500. The mean age of participants was in the early 50s. Both the prevalence and incidence rates of bilateral HFHL increased sharply from the 60s and were higher in males than in females across generations; the prevalence was < 4% in the early 50s, reaching 46.5% and 20.2% in males and females, respectively, in their 70s. Incidence rates were 10.8 and 2.1, respectively, in the 50s, increasing to 106.7 and 43.5, respectively, in the 80s. Age-standardized prevalence slightly decreased from 2014 to 2020 in both sexes.

**Conclusions:**

Both the prevalence and incidence rates of bilateral HFHL increased dramatically from the 60s and were higher in males than in females across generations. Age-standardized prevalence slightly decreased during the study period. This study is valuable because of the limited number of studies on hearing loss in Japan. However, most participants were considered to have high socioeconomic status, and further research targeting Japanese individuals is warranted.

## Introduction

Hearing loss is a significant global public health concern; it has negative effects, particularly on older adults, contributing to social isolation and depression through communication difficulties ^(1), (2)^, dementia ^(2), (3), (4)^, increasing frailty or falls ^(5), (6)^, and increased social costs ^(7), (8)^. It has also been reported as an independent risk factor for elevated mortality ^(9)^. Moreover, the worldwide prevalence of hearing loss, encompassing all severity levels, is approximately 20% and is expected to rise further with the aging population ^(10)^. Thus, hearing loss poses substantial challenges for both individuals and society.

Hearing ability begins to decline bilaterally at high frequencies (high-frequency hearing loss [HFHL]) as early as the 30s and 40s, influenced by various factors such as sex and age ^(11), (12)^. The prevalence of hearing loss begins to rise in individuals in their 50s, with a more pronounced increase in the 60s. After the 80s, the prevalence exceeded 50% ^(10), (12), (13)^. Males consistently exhibit higher prevalence than females. This trend is common across different regions, although the prevalence varies by region ^(12), (13)^. Regarding age-standardized prevalence, globally, it increased from 1990 to 2019, but the change varied by degree of hearing loss and region ^(14)^. To assess hearing loss, the pure-tone average of 0.5, 1, 2, and 4 kHz thresholds in the better hearing ear has been commonly used, following the World Health Organization criteria ^(10)^. Therefore, research on the prevalence of HFHL, which is considered an early stage of hearing loss, is limited.

Limited data on hearing loss are available from Japan, although the country has particularly high aging rates ^(15), (16)^. Uchida et al. reported that the prevalence of hearing loss (pure-tone average ≥ 25 dB) in Japanese individuals aged 65-69 years was 43.7% in males and 27.7% in females, increasing to 84.3% in males and 73.3% in females among those aged ≥ 80 years ^(16)^. To our knowledge, however, no studies have reported incidence rates and age-standardized prevalence of hearing loss in Japan ^(14), (17), (18), (19)^. Even when examined across regions or classifications, including Japan, the age-standardized prevalence remained largely unchanged in high-income Asia Pacific regions; however, it increased in high socio-demographic index areas. This discrepancy suggests that it is difficult to estimate the specific change in age-standardized prevalence in Japan. Further research on hearing loss in Japan, a country with high aging rates, is needed.

Japan has different types of health check-up examinations. Hearing tests are usually performed during regular health check-up examinations and the Comprehensive Health Check-up System (*Ningen Dock*), using frequencies 1 kHz and 4 kHz; employers are required to have their employees undergo regular health check-up examinations at least once a year ^(20), (21), (22)^. The *Ningen Dock*, although not mandatory, is a comprehensive health examination system for individuals who voluntarily seek to monitor their health and achieve early disease detection through more extensive tests in addition to those included in the regular health check-up examination ^(23), (24), (25)^. Some examinees opt for the *Ningen Dock* as an alternative to the regular health check-up examination.

This study aimed to describe the prevalence, age-standardized prevalence, and incidence rates of bilateral HFHL among the Japanese population, using 4-kHz data from health check-up examinations.

## Materials and Methods

### Study design and data source

**Figure 1.**
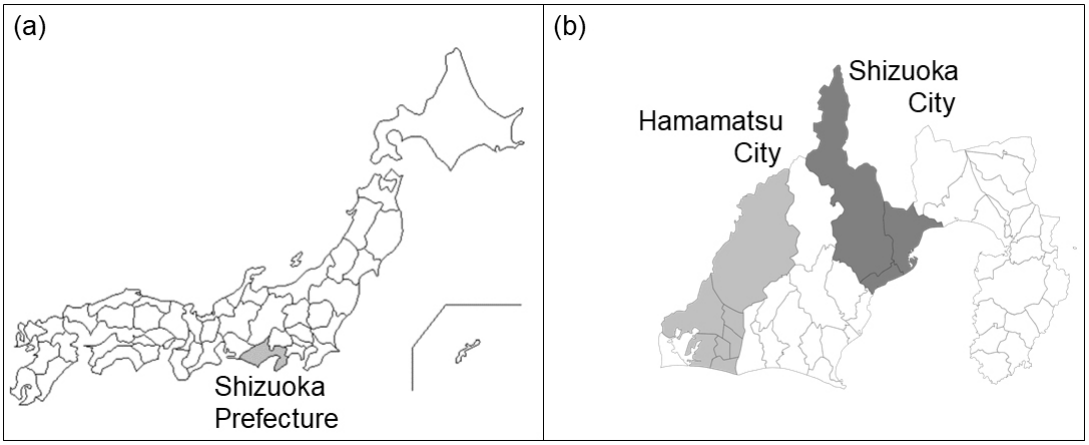
Location map. Shizuoka Prefecture in the map of Japan (a), and (b) Hamamatsu City and Shizuoka City in the Shizuoka Prefecture. Five facilities of the Seirei Health Care Division are located in the two cities in Shizuoka Prefecture.

This descriptive study utilized data from health check-up examinations. The data were collected from examinations conducted by the Seirei Health Care Division (hereinafter, referred to as Seirei) between April 2014 and March 2021. Seirei comprises five facilities: three located in Hamamatsu City and two in Shizuoka City, both within Shizuoka Prefecture (Figure 1). Hamamatsu City, one of the 20 government ordinance-designated cities, has the largest population in Shizuoka Prefecture. Shizuoka City serves as the prefectural capital of Shizuoka Prefecture. A cross-sectional study using FY2020 data was applied for prevalence analysis. Repeated cross-sectional studies between FY2014 and FY2020 were applied for age-standardized prevalence analyses. A dynamic cohort design was applied for the incidence rate analysis. Further details are provided in the following Statistical Analysis section.

### Pure-tone audiometry in this study

Pure-tone audiometry was performed as part of a regular health check-up examination, with screening thresholds set at 30 dB at 1 kHz and 40 dB at 4 kHz for each ear ^(20), (21)^. Individuals who failed to hear at these thresholds were sometimes referred to otolaryngologists, depending on the degree of hearing loss and results of previous screening tests. At Seirei, screening test results at regular health check-up examinations were recorded as a combination of two frequencies (1 kHz and 4 kHz); this made it unclear at which frequency hearing ability declined. Only a very small proportion of the participants undergoing regular health check-up examinations had the results separately at each frequency, partly due to contractual arrangements with their employers. In the *Ningen Dock* at Seirei*,* the same screening test was conducted, but the results were recorded separately for each frequency. Hearing tests were conducted in a soundproof room at Seirei for the *Ningen Dock,* and for regular health check-up examination participants, either in a soundproof room or in an environment with noise levels below 40 dB.

### Participants

Participants who underwent bilateral screening audiometry at least once between April 2014 and March 2021 were included in this study. Those eligible for the noise health check-up examination and those who did not have separate results for 1 kHz and 4 kHz were excluded.

### Statistical analysis

Participant characteristics were summarized using means with 95% confidence intervals (CIs) for continuous variables, such as examination data, and proportions with 95%CI for categorical variables, such as questionnaire data in fiscal year (FY) 2014 and in FY2020. When participants had the *Ningen Dock* or regular health check-up examination twice or more in the same FY, the first data of the FY were used.

The outcome was defined as inaudibility of 40 dB at 4 kHz in both ears, termed bilateral HFHL in this study. Prevalence was calculated by sex and age group, using FY2020 data, with age groups classified as < 35, 35-39, 40-44, 45-49, 50-54, 55-59, 60-64, 65-69, 70-74, 75-79, and ≥ 80 years. Age-standardized prevalence from FY2014 to FY2020 was calculated by the direct method, using the 2015 Japan Standard Population ^(26), (27)^.

For incidence rates, participants who underwent their first health check-up examination with a hearing test between FY2014 and FY2019, bilaterally heard 40 dB at 4 kHz at that time, and had at least one additional hearing test thereafter, were analyzed using the person-year method. It was assumed that bilateral HFHL onset occurred mid-year between the last year when bilateral HFHL was not detected and the first year of detection. A sensitivity analysis was additionally conducted, assuming that the onset occurred either just after the last year when bilateral HFHL was not detected or just before the first year of detection. For example, if the last year when bilateral HFHL was undetected was FY2016 and the first year of detection was FY2020, the onset was assumed to be FY2018, equating to 4.0 person-years, with sensitivity analysis ranging from 2.0 to 6.0 person-years. A complete case analysis was planned, as the number of missing age and sex data was anticipated to be very small.

Statistical analyses were performed using Excel 2019 (Microsoft Corp., Redmond, WA, USA) and SPSS Statistics (version 26.0; IBM Corp., Armonk, NY, USA).

### Ethics

This study was performed in accordance with the Declaration of Helsinki and was approved by the Clinical Research Ethics Committee of Hamamatsu University School of Medicine (Approval No. 23-109) and the Kyoto University Graduate School and Faculty of Medicine Ethics Committee (R4202). This study used an opt-out system, and informed consent was waived. Participants were given the opportunity to refuse participation in the study and withdraw their consent to participate.

## Results

### Participant characteristics

More than 95% of the participants underwent the *Ningen Dock,* with approximately 60% being male. The mean age of participants was in the early 50s in both FY2014 and FY2020. The proportion of current smokers decreased from 17.2% in FY2014 to 14.4% in FY2020, and the proportion of daily drinkers decreased from 16.2% to 14.2%. However, the proportion of individuals who gained ≥ 10 kg since the age of 20 and those under treatment for non-communicable diseases increased during this period (Table 1).

**Table 1.** Characteristics of Participants in FY2014 and FY2020.

Participant Characteristics	In FY2014 (N = 55771)	In FY2020 (N = 62634)
Examinees undergoing the *Ningen Dock* (n, [%])	53497 (95.9)	61558 (98.3)
Men (n, [%])	35449 (60.2)	36351 (58.0)
Age (yrs), mean (SD)	52.0 (11.1)	53.0 (11.5)
Body mass index (kg/m^2^), mean (SD)	22.7 (3.4)	23.1 (3.6)
Systolic blood pressure (mmHg), mean (SD)	115.7 (14.8)	116.6 (14.6)
Fasting blood glucose (mg/dL), mean (SD)	97.1 (16.4)	99.7 (17.0)
LDL-C (mg/dL), mean (SD)	128.6 (30.7)	127.2 (30.2)
Current regular smoker^a^ (n, [%])	9478 (17.0)	9013 (14.4)
Drinking every day (n, [%])	9152 (16.4)	8677 (14.2)
Weight gain of ≥10 kg since age 20 years (n, [%])	19510 (35.0)	23141 (38.0)
Under treatment of HT (n, [%])	8501 (15.2)	10656 (17.0)
Under treatment of DM (n, [%])	2097 (3.8)	2828 (4.5)
Under treatment of dyslipidemia (n, [%])	6504 (11.7)	9339 (14.9)

DM: diabetes mellitus; FY: fiscal year; HT: hypertension; LDL-C: low density lipoprotein cholesterol; *Ningen Dock*: Comprehensive Health Check-up System in Japan; SD: standard deviation. ^a^“Current regular smoker” refers to those who have smoked a total of over 100 cigarettes or have smoked over a period of 6 months and have been smoking over the past month. The number of missing values was as follows, starting from the top of the table (in 2014/in 2020). Items not mentioned have no missing data; body mass index (17/0), systolic blood pressure (18/8), fasting blood glucose (264/341), LDL-C (115/81), current regular smoker (23/51), drinking daily and bodyweight gain ≥10 kg since age 20 (22/1,708), and under treatment of HT, DM, and dyslipidemia (22/49).

### Prevalence and age-standardized prevalence

**Figure 2.**
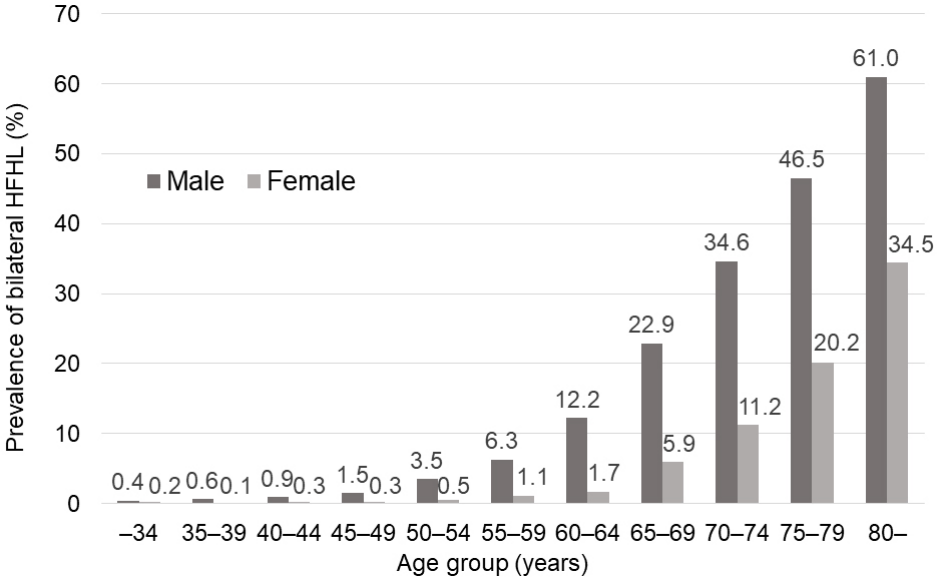
Prevalence of bilateral high-frequency hearing loss by age group in FY2020 (N = 62,634).

**Figure 3.**
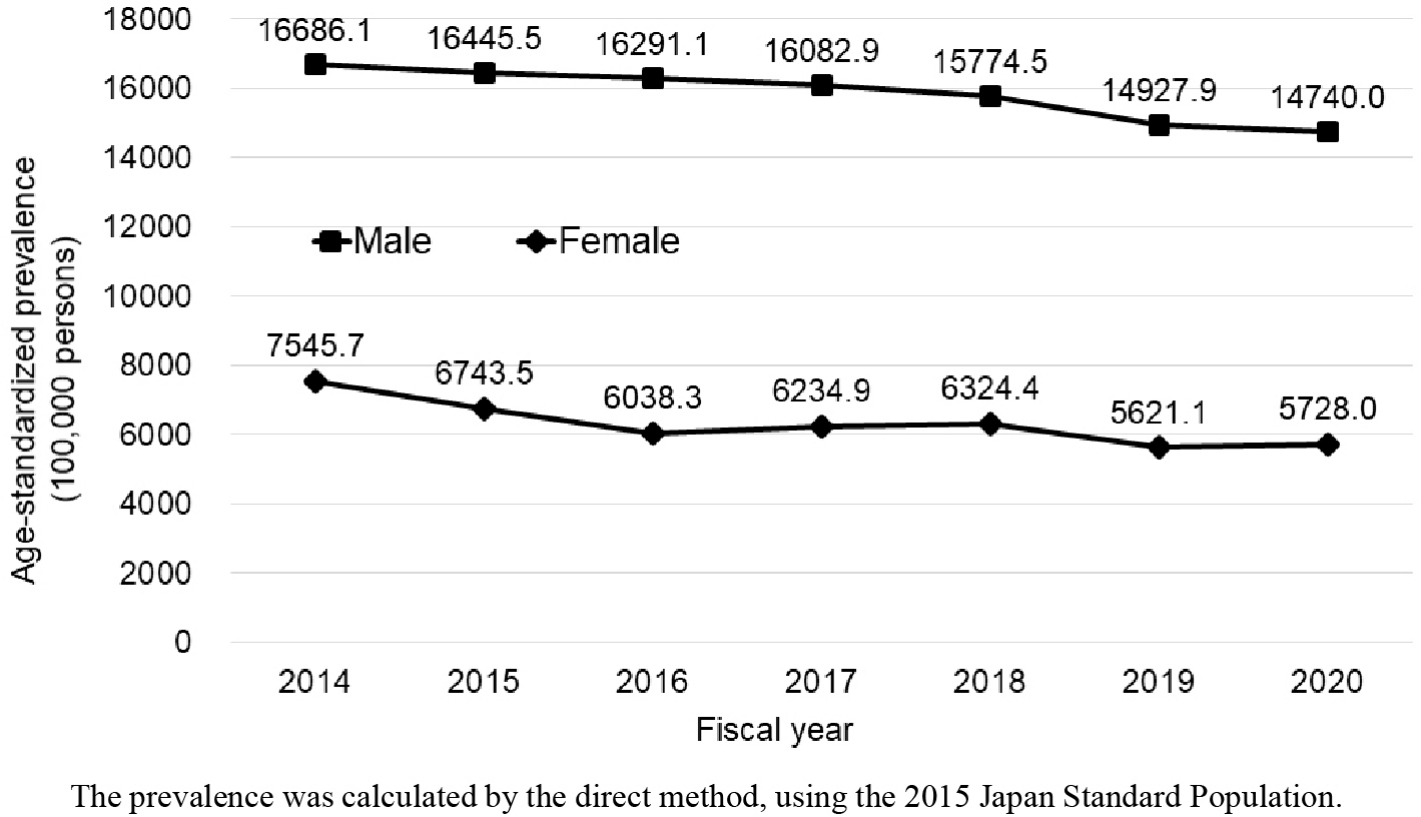
Age-standardized prevalence of bilateral high-frequency hearing loss between FY2014 and FY2020.

In 2020, 62,634 participants were included in the analysis of bilateral HFHL prevalence. The prevalence was consistently higher in males than in females across all age groups. For both sexes, the prevalence significantly increased in individuals in their 60s. In males, prevalence was 3.5% in their early 50s, 12.2% in their early 60s, and 46.5% in their 70s. In females, prevalence was 0.5% in their early 50s, 1.7% in their early 60s, and 20.2% in their late 70s (Figure 2 and Table 2). The age-standardized prevalence per 100,000 individuals decreased from FY2014 to FY2020 for both sexes: from approximately 17,000 to 15,000 in males and from approximately 7,500 to 5,700 in females (Figure 3). The decrease was statistically significant in both sexes, according to the regression analysis results (Table S1). There was no missing data on the sex and age of participants with bilateral HFHL.

**Table 2.** Prevalence of Bilateral High-Frequency Hearing Loss (HFHL)^a^ in 2020.

Age group	Males		Females		p-Value^b^
(years)	(%)	(n/N)	(%)	(n/N)	
≤34	0.4	(5/1,159)	0.2	(3/1,417)	0.521
35-39	0.6	(14/2,360)	0.1	(1/1,861)	0.008
40-44	0.9	(41/4,506)	0.3	(9/3,493)	<0.001
45-49	1.5	(88/5,802)	0.3	(12/4,557)	<0.001
50-54	3.5	(199/5,738)	0.5	(21/4,252)	<0.001
55-59	6.3	(360/5,702)	1.1	(43/3,811)	<0.001
60-64	12.2	(556/4,546)	1.7	(52/3,035)	<0.001
65-69	22.9	(683/2,989)	5.9	(116/1,972)	<0.001
70-74	34.6	(772/2,232)	11.2	(144/1,290)	<0.001
75-79	46.5	(425/914)	20.2	(87/430)	<0.001
≥80	61.0	(246/403)	34.5	(57/165)	<0.001

^a^HFHL is defined as a hearing loss of 4,000 Hz at 40 dB in this study. Chi-square tests were used to examine the association between sex and prevalence of bilateral HFHL. p-Values of <0.05 were considered statistically significant.

### Incidence rates

**Figure 4.**
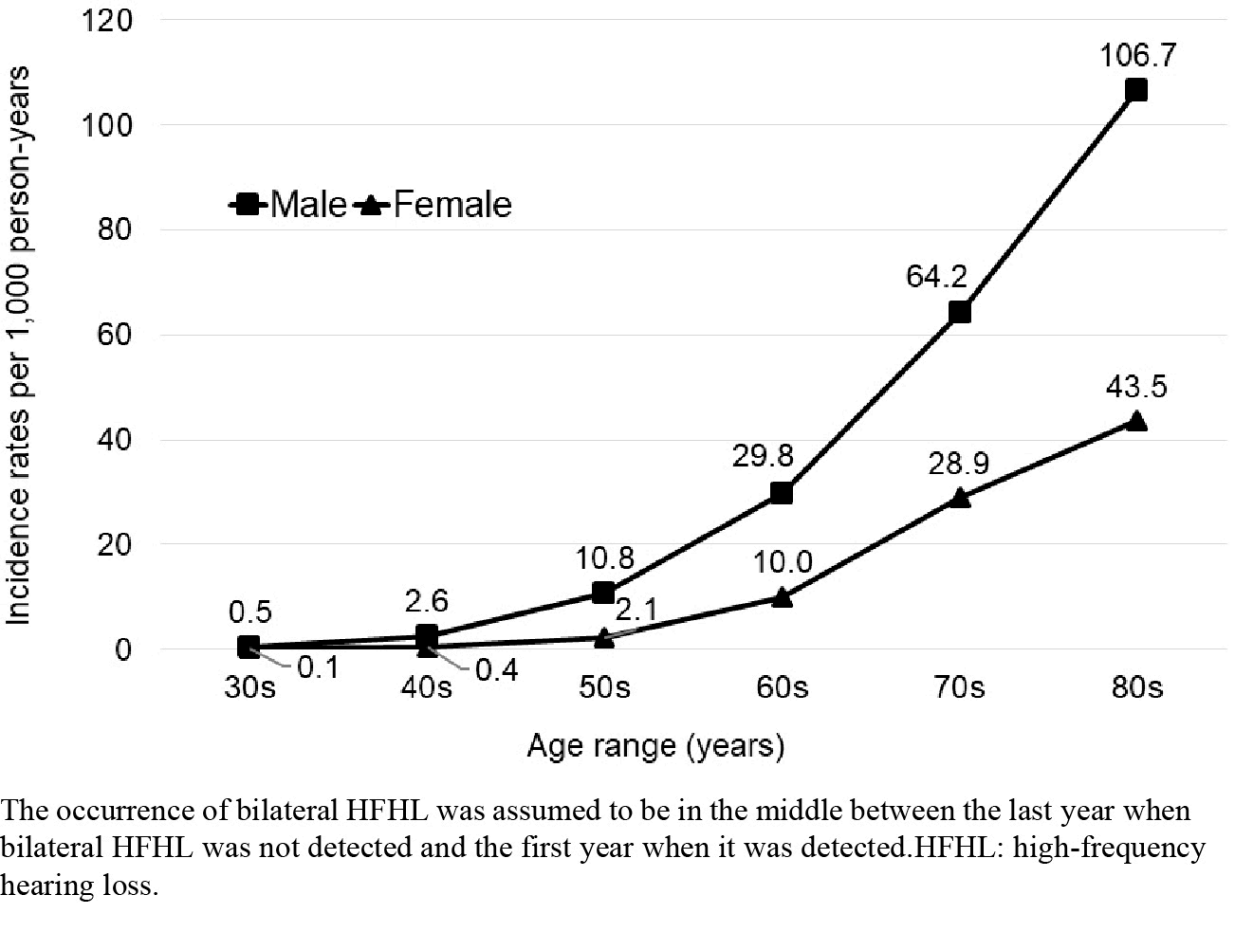
Incidence rates of bilateral HFHL (N = 77,876).

In total, 77,876 participants (44,135 males and 33,741 females) who bilaterally heard 40 dB at 4 kHz at the time of the first hearing test between FT2014 and FY2019 were included in the analysis of incidence rates for bilateral HFHL. The number of participants retained in follow-up by year of inclusion and follow-up year is shown in Table S2. There were no missing data regarding sex or age. Figure 4 presents the incidence rates for each generation by sex. The incidence rates were consistently higher in males than in females across generations. The incidence rate per 1,000 person-years increased dramatically after the 60s in both sexes. In participants in their 50s, the incidence rates per 1,000 person-years were 10.8 for males and 2.1 for females, whereas the rates were 106.7 for males and 43.5 for females in their 80s (Table 3). Sensitivity analysis showed similar results (Table S3 and S4).

**Table 3.** Incidence Rate per 1,000 Person-Years of Bilateral High-Frequency Hearing Loss (HFHL)^a^ between 2014 and 2020.

Age range	Males				Females				
(years)	Incidence rate^b^	Incidence^c^	n^d^	Person-years	Incidence rate^b^	Incidence^c^	n^d^	Person-years	*p*-Value^e^
30s	0.5 (0.227-0.819)	12	6,508	22,939	0.1 (0.000-0.251)^f^	2	5,181	19,044.5	0.04
40s	2.6 (2.219-3.012)	167	15,418	63,846	0.4 (0.254-0.635)	21	11,425	47,240	<0.001
50s	10.8 (9.900-11.623)	593	13,011	55,103	2.1 (1.676-2.573)	86	9,642	40,488	<0.001
60s	29.8 (27.898-31.874)	803	6,998	26,909.5	10.0 (8.647-11.293)	216	5,424	21,665	<0.001
70s	64.2 (57.605-80.874)	337	1,501	5,246	28.9 (23.613-34.156)	112	1,116	3,877.5	<0.001
80s	106.7 (61.379-152.105)	19	65	178	43.5 (14.012-72.945)	8	54	184	0.04

^a^HFHL is defined as hearing loss of 4,000 Hz at 40 dB in this study. ^b^“Incidence rate” refers to incidence rate per 1,000 person-years, and values in parentheses represent the results of 95% confidence intervals. ^c^“Incidence” refers to the number of persons with bilateral HFHL detected during the follow-up period ^d^“n” refers to the number of persons tracked. ^e^χ^2^ tests were used to examine the association between sex and incidence rate. ^f^If the lower limit of the 95% confidence interval was calculated as less than 0, it was set as 0.000.

## Discussion

The prevalence and incidence rates of bilateral HFHL were higher in males than in females across generations, with a dramatic increase observed after the 60s. One in 10 males in their 80s developed bilateral HFHL every year. The age-standardized prevalence slightly decreased from FY2014 to FY2020 for both sexes.

The increase in prevalence and incidence rates with age, and the higher prevalence and incidence rates in males than in females, are consistent with previous studies, although there is limited research on incidence rates evaluated by person-years. Both prevalence and incidence rates began increasing significantly after the age of 60 and were higher in males than in females across all generations ^(2), (3), (10), (17)^. This study supports evidence that these trends are consistent across various regions.

The age-standardized prevalence slightly decreased from FY2014 to FY2020 in both males and females in this study. This aligns with previous studies on the age-standardized prevalence of hearing loss over moderate levels, considering that inaudibility of 40 dB at 4 kHz in this study corresponds to hearing loss over moderate levels ^(14)^. The decline in age-standardized prevalence of bilateral HFHL can be attributed to several factors: aging, noise exposure, and ototoxic medications are the primary causes of hearing loss ^(28)^. In Japan, fewer individuals work in noisy environments because of industrial structure changes, improvements in occupational health, and easier access to medical care through the universal health insurance system ^(22), (25), (29), (30)^. In particular, participants who underwent the *Ningen Dock* were highly health-conscious. Indeed, the proportion of participants receiving treatment for hypertension or diabetes mellitus increased, and smoking rates decreased from FY2014 to FY2020. As smoking, hypertension, and diabetes mellitus have been identified as risk factors of hearing loss, these changes likely influenced the decline in age-standardized prevalence ^(20), (30), (31), (32), (33), (34), (35)^.

This study has some limitations. First, the outcome of hearing loss differed from previous studies. Most previous studies used the pure-tone average of threshold at four frequencies (0.5, 1, 2, and 4 kHz) based on World Health Organization criteria, which are considered to be closely related to the daily inconvenience caused by hearing loss ^(36)^. The outcome of the incidence rates was subjective, although there is limited research on incidence rates calculated by person-years ^(17)^. The differences in outcomes, in addition to regional or ethnic differences, make simple comparisons difficult. Hearing loss typically begins at high frequencies, and individuals with inaudibility of 40 dB at 4 kHz may not feel inconvenience ^(11), (12), (37), (38)^. The outcome of this study can detect hearing loss at an earlier stage and can be useful in research on hearing loss. Second, the outcome in this study may include the 4-kHz notch (c5-dip) in noise-induced hearing loss. Participants eligible for the noise health check-up examination were excluded, but those who work in noisy environments just below the environmental measurement standard, or individuals with noise-induced hearing loss due to audio equipment, could be included ^(10)^. Increasing concerns about noise-induced hearing loss from audio equipment may affect future prevalence and incidence rates. Third, the causes of bilateral HFHL were not considered. Aging, noisy environments, and ototoxic medications are the primary causes of bilateral HFHL ^(28)^. Different populations exposed to varying noise levels or medications could yield different results. Fourth, the participants have some selection biases; most participants in this study underwent the *Ningen Dock*. They likely had a high socioeconomic status because of its high cost ^(23)^. Association between hearing loss and socioeconomic status has been reported, and the results in this study may be underestimated ^(32), (33), (34), (35), (39), (40)^. Moreover, the participants, especially the elderly participants, had healthy user bias. Therefore, generalization to the Japanese population is challenging. Nevertheless, the findings from this study involving approximately 78,000 individuals without intervention provide valuable insights, given the limited evidence in Japan.

### Conclusions

We described the prevalence, age-standardized prevalence, and incidence rates of bilateral HFHL among individuals undergoing the *Ningen Dock* in Japan, where limited evidence on hearing loss exists despite high aging rates. The results indicated that the prevalence and incidence rates were consistently higher in males than in females across generations, with a significant increase in individuals in their 60s and older. Additionally, the age-standardized prevalence showed a slight decrease in both sexes from FY2014 to FY2020. This study is significant in the field of hearing loss in Japan, a country with high aging rates, due to the limited data available on hearing loss in Japan.

## Article Information

### Acknowledgments

The authors are grateful to Professor Dr. Akira Takagi, Professor in the Department of Hearing & Language Studies, Shizuoka Graduate University of Public Health, for his advice on the methods and interpretation of the results of this study. We are also grateful to the Seirei Health Care Division for data provision and, all the staff, including Dr. Uchino Asuka at the Seirei Health Care Division for data provision and advice regarding data handling and interpretation.

### Author Contributions

Conceptualization: Yuri Akamatasu, Yoshitaka Nishikawa, Mayumi Toyama, Yoshimitsu Takahashi, Hiroshi Nakanishi, Kiyoshi Misawa, Toshiyuki Ojima, and Takeo Nakayama, Data curation: Yuri Akamatsu and Toshiyuki Ojima, Formal Analysis: Yuri Akamatsu, Yoshitaka Nishikawa, Mayumi Toyama, Yoshimitsu Takahashi, and Takeo Nakayama, Funding acquisition: Takeo Nakayama, Investigation: Yuri Akamatsu, Yoshitaka Nishikawa, Mayumi Toyama, Yoshimitsu Takahashi, Hiroshi Nakanishi, and Takeo Nakayama, Methodology: Yuri Akamatsu, Yoshitaka Nishikawa, Mayumi Toyama, Yoshimitsu Takahashi, Hiroshi Nakanishi, and Takeo Nakayama, Project administration: Yuri Akamatsu, Yoshitaka Nishikawa, Mayumi Toyama, Yoshimitsu Takahashi, and Takeo Nakayama, Resources: Yuri Akamatsu and Toshiyuki Ojima, Software: Yuri Akamatsu and Toshiyuki Ojima, Supervision: Takeo Nakayama, Validation: N.A., Visualization: Yuri Akamatsu, Yoshitaka Nishikawa, Mayumi Toyama, Yoshimitsu Takahashi, and Takeo Nakayama, Writing - original draft: Yuri Akamatsu and Yoshitaka Nishikawa, Writing - review & editing: Yuri Akamatsu, Yoshitaka Nishikawa, Mayumi Toyama, Yoshimitsu Takahashi, . Hiroshi Nakanishi, Kiyoshi Misawa, Toshiyuki Ojima, and Takeo Nakayama. All authors read and approved the final manuscript.

### Conflicts of Interest

TN received grants from I&H Co., Ltd, Cocokarafine Group Co., Ltd, Konica Minolta, Inc., and NTT DATA, and did consulting fees from Otsuka Pharmaceutical Co., Takeda Pharmaceutical Co., Johnson & Johnson K.K., and Nippon Zoki Pharmaceutical Co., Ltd., and has received payments or honoraria for lectures, presentations, speakers bureaus, manuscript preparation, or educational meetings from Pfizer Japan Inc., MSD K.K., Chugai Pharmaceutical Co., Takeda Pharmaceutical Co., Janssen Pharmaceutical K.K., Boehringer Ingelheim International GmbH, Eli Lilly Japan K.K., Maruho Co., Ltd., Mitsubishi Tanabe Pharma Co., Novartis Pharma K.K., Allergan Japan K.K., Novo Nordisk Pharma Ltd., TOA EIYO Ltd., Dentsu co., ONO PHARMACEUTICAL CO., LTD., GSK plc, Alexion Pharmaceuticals, Inc., Cannon Medical Systems Co., Araya, and AbbVie Inc.

TN also assumes leadership or fiduciary role in other board, society, committee or advocacy group, paid or unpaid Chair, Expert Panel for the 4th Term of the Specific Health Examinations and Health Guidance, Ministry of Health, Labor and Welfare, Japan Chair, Review and Evaluation Committee for the Large-Scale Examination Project on Prevention and Health Promotion, Ministry of Health, Labor and Welfare, Japan Program Supervisor, Infrastructure Project for Social Implementation of Health Care, Japan Agency for Medical Research, Ministry of Economy, Trade and Industry, Japan Chairman, Steering Committee of e-Health Net, Ministry of Health, Labor and Welfare, Japan Program Supervisor, Infrastructure Development Project for Social Implementation of Health Care, Japan Agency for Medical Research and Development, Chairman, Advisory and Review Committee on Registry Information Provision, National Cancer Center National Cancer, Japan Affiliate Member (25th), Science Council of Japan; Chair, Minds Guideline Center, Japan Council for Quality Health Care; Trustee, General Incorporated Association Life Data Initiative, Japan; Trustee, General Incorporated Association Patients Association Information Center, Japan; Trustee, General Incorporated Association Research Institute of Healthcare Data Science, Japan; Chair, NPO Evidence Based Healthcare Council, Japan; Chair, NPO Japan Medical and Scientific Communicators Association; and Vice Chair, NPO DIPEX Japan. TN received donation from Cancerscan and YUYAMA CO., LTD., and YN received donation from Datack Inc. and Cancerscan Inc.

YT is employed through a joint research fund between Kyoto University and HealthTech Laboratory Inc.

### IRB Approval Code and Name of the Institution

This study was performed in accordance with the Declaration of Helsinki and approved by the Clinical Research Ethics Committee of Hamamatsu University School of Medicine (Approval No. 23-109) and Kyoto University Graduate School and Faculty of Medicine, Ethics Committee (R4202). In this study, an opt-out system was used to obtain consent from participants. The participants had the opportunity to refuse to participate in the study and withdraw their consent to participate.

## Supplement

Supplementary Material
