# Supplementary material for: Prevalence, Age-Standardized Prevalence, and Incidence Rates of Bilateral High-Frequency Hearing Loss among Japanese Individuals Undergoing Comprehensive Health Checkup System (Ningen Dock) from 2014 to 2020: A Descriptive Study
Source: JMA J. 2025 Dec 19;9(1):115–23. doi: 10.31662/jmaj.2024-0328 (PMC12889015; doi:10.31662/jmaj.2024-0328)
Supplement: Supplementary Material [file 2433-3298-9-1-0115-s001.pdf]

Supplementary Table 1.
Regression Analysis of Changes in Age-Standardized Prevalence from 2014 to 2020.

	Regression Coefficient	(95% Confidence Interval)	*p*-value	R^2
Male	-335.37	(-442.14, -228.60)	<0.001	0.93
Female	-264.71	(-439.23, -90.20)	0.011	0.75

Supplementary Table 2. The Number of Participants Retained in Follow-up by Year of Inclusion and Follow-up Year.

Year of Inclusion	Year of Follow Up						
	FY2014	FY2015	FY2016	FY2017	FY2018	FY2019	FY2020
FY2014	44216	44216	40757	37992	35426	32426	27606
FY2015	-	10401	10401	9541	8560	7526	5688
FY2016	-	-	8877	8877	8129	7143	5652
FY2017	-	-	-	5674	5674	4922	3898
FY2018	-	-	-	-	4924	4924	3911
FY2019	-	-	-	-	-	3784	3784

Supplementary Table 3. Sensitivity Analysis of Bilateral High-Frequency Hearing Loss (HFHL) Incidence Rate per 1,000 Person-years, Assuming the Onset Was Just Before the First Year When the Bilateral HFHL was Detected (2014–2020).

Age Range (years)	Male				Female				
	Incidence Rate†	Incidence‡	n§	Person-years	Incidence Rate†	Incidence‡	n§	Person-years	*p* value⁄⁄
30s	0.523	12	6,508	22,947	0.105	2	5,181	19,047	0.04
40s	2.612	167	15,418	63,947	0.444	21	11,425	47,255	<0.001
50s	10.696	593	13,011	55,442	2.122	86	9,642	40,537	<0.001
60s	29.347	803	6,998	27,362	9.911	216	5,424	21,794	<0.001
70s	61.903	337	1,501	5,444	28.369	112	1,116	3,948	<0.001
80s	101.064	19	65	188	42.328	8	54	189	0.03

* HFHL is defined as hearing loss of 4,000 Hz at 40 dB in this study.

† "Incidence rate" refers to incidence rate per 1,000 person-years.

‡ "Incidence" refers to the number of persons with bilateral HFHL detected during the follow-up period.

§ "n" refers to the number of persons tracked.

⁄⁄ Chi-square tests were used to examine the association between sex and incidence rate.

Supplementary Table 4. Sensitivity Analysis of Bilateral High-Frequency Hearing Loss (HFHL) Incidence Rate per 1,000 Person-years, Assuming the Onset Was Just After the Last Year When the Bilateral HFHL Was Not Detected (2014–2020).

Age Range (years)	Males				Female				
	Incidence Rate†	Incidence‡	n§	Person-years	Incidence Rate†	Incidence‡	n§	Person-years	*p* value⁄⁄
30s	0.523	12	6,508	22,931	0.105	2	5,181	19,042	0.04
40s	2.620	167	15,418	63,745	0.445	21	11,425	47,225	<0.001
50s	10.828	593	13,011	54,764	2.127	86	9,642	40,439	<0.001
60s	30.351	803	6,998	26,457	10.03	216	5,424	21,536	<0.001
70s	66.759	337	1,501	5,048	29.419	112	1,116	3,807	<0.001
80s	113.095	19	65	168	44.693	8	54	179	0.03

* HFHL is defined as hearing loss of 4,000 Hz at 40 dB in this study.

† "Incidence rate" refers to incidence rate per 1,000 person-years.

‡ "Incidence" refers to the number of persons with bilateral HFHL detected during the follow-up period.

§ "n" refers to the number of persons tracked.

⁄⁄ Chi-square tests were used to examine the association between sex and incidence rate.
